# Association of salivary alpha-amylase with anxiety and stress in nursing professionals*

**DOI:** 10.1590/1518-8345.4859.3468

**Published:** 2021-08-30

**Authors:** Sergio Valverde Marques dos Santos, Luiz Almeida da Silva, Fábio de Souza Terra, Adriele Vieira de Souza, Foued Salmen Espindola, Maria Helena Palucci Marziale, Renata Roland Teixeira, Maria Lucia do Carmo Cruz Robazzi

**Affiliations:** 1 Universidade de São Paulo, Escola de Enfermagem de Ribeirão Preto, PAHO/WHO Collaborating Centre for Nursing Research Development, Ribeirão Preto, SP, Brazil.; 2 Universidade do Estado de Minas Gerais, Campus Passos, Passos, MG, Brazil.; 3 Universidade Federal de Catalão, Departamento de Enfermagem, Catalão, GO, Brazil.; 4 Universidade Federal de Alfenas, Escola de Enfermagem, Alfenas, MG, Brazil.; 5 Universidade Federal de Uberlândia, Instituto de Biotecnologia, Uberlândia, MG, Brazil.

**Keywords:** Nursing, Occupational Health, Psychological Stress, Anxiety, Biomarker, Alpha-Amylases, Enfermagem; Saúde do Trabalhador, Estresse Psicológico, Ansiedade, Biomarcador, Alfa-Amilases, Enfermería, Salud Laboral, Estrés Psicológico, Ansiedad, Biomarcador, Alfa-Amilasas

## Abstract

**Objective:**

to assess if changes in salivary alpha-amylase activity are associated with anxiety and stress among hospital nursing professionals and whether anxiety and stress are associated with sociodemographic, epidemiological, and occupational factors.

**Method:**

cross-sectional, quantitative study, carried out with 210 nursing professionals from a hospital. For data collection, we used a questionnaire to characterize workers, Beck’s Anxiety Inventory, Lipp’s Stress Symptoms Inventory for Adults and samples and saliva samples collected in work shifts. The data were analyzed descriptively and inferentially using the software Statistical Package for the Social Science and GraphPad Prism.

**Results:**

most professionals experienced stress and anxiety. The variables age group, number of children, use of medication and workload were associated with anxiety; age group, smoking and medication use were associated with stress. An increase in the *salivary alpha-amylase activity* was observed in the middle of the work shift. Professionals who had stress and anxiety had significant changes in alpha-amylase in the night shift.

**Conclusion:**

changes in salivary alpha-amylase were associated with anxiety and stress among nursing professionals, indicating that this enzyme can be a possible biomarker of anxiety and stress in workers.

## Abstract (pt)

**Objetivo:**

avaliar se a alteração na atividade da alfa amilase salivar está associada à ansiedade e ao estresse dos profissionais de enfermagem hospitalar e, se a ansiedade e o estresse estão associados aos fatores sociodemográficos, epidemiológicos e laborais.

**Método:**

estudo transversal, quantitativo, desenvolvido com 210 profissionais de enfermagem de uma instituição hospitalar. Para a coleta de dados, utilizouse um questionário de caracterização dos trabalhadores, o Inventário de Ansiedade de Beck, o Inventário de Sintomas de Stress para Adultos de Lipp e as amostras de saliva coletadas durante os turnos de trabalho. Os dados foram analisados de forma descritiva e inferencial utilizando os *softwares Statistical Package for the Social Science* e *GraphPad Prism*.

**Resultados:**

a maioria dos profissionais apresentou estresse e ansiedade. As variáveis faixa etária, quantidade de filhos, uso de medicamentos e carga horária de trabalho estavam associadas à ansiedade; faixa etária, tabagismo e uso de medicamentos estavam associadas ao estresse. Observouse um aumento da atividade da alfa amilase salivar no meio do turno de trabalho. Os profissionais que tinham estresse e ansiedade apresentaram alteração significativa da alfa amilase no turno da noite.

**Conclusão:**

as alterações na atividade da alfa amilase estavam associadas à ansiedade e ao estresse dos profissionais de enfermagem, demonstrando que essa enzima pode ser um possível biomarcador de ansiedade e estresse em trabalhadores.

## Abstract (es)

**Objetivo:**

evaluar si la alteración en la actividad de la alfa amilasa salival está asociada a la ansiedad y al estrés de los profesionales de enfermería hospitalaria y, si la ansiedad y el estrés están asociados a factores sociodemográficos, epidemiológicos y laborales.

**Método:**

estudio transversal, cuantitativo, desarrollado en 210 profesionales de enfermería, en una institución hospitalaria. Para la recogida de datos, se utilizó un cuestionario de caracterización de los trabajadores, el Inventario de Ansiedad de Beck, el Inventario de Síntomas de *Stress* para Adultos de Lipp y las muestras de saliva recogidas durante los turnos de trabajo. Los datos fueron analizados de forma descriptiva e inferencial utilizando los *softwares Statistical Package for the Social Science* y *GraphPad Prism*.

**Resultados:**

la mayoría de los profesionales presentó estrés y ansiedad. Las variables intervalo etario, cantidad de hijos, uso de medicamentos y carga horaria de trabajo estaban asociadas a la ansiedad; el intervalo etario, el tabaquismo y el uso de medicamentos estaban asociados al estrés. Se observó un aumento de la actividad de la alfa amilasa salival en los ambientes de los turnos de trabajo. Los profesionales que tenían estrés y ansiedad presentaron alteración significativa de la alfa amilasa en el turno de la noche.

**Conclusión:**

las alteraciones en la actividad de la alfa amilasa estaban asociadas a la ansiedad y al estrés de los profesionales de enfermería, demostrando que esa enzima puede ser un posible biomarcador de ansiedad y estrés en trabajadores.

## Introduction

Mental disorders have been considered one of the major health conditions current in the workplace. These disorders have led to job losses, disabilities, and decreased productivity in many work fields, with significant economic impact. The estimated cost for the global economy is US$ 1 trillion *per* year in lost productivity^(1)^. In Brazil, mental illness is the third leading cause of incapacity for work; disorders triggered by stress, anxiety and depression caused 79% of work leaves between 2012 and 2016^(2)^.

In the health field the prevalence of mental disorders is high, leading to risks for service users, especially when professionals show symptoms of psychological changes. Nursing workers are one of the categories that most suffer from mental disorders, due to their work process, characterized by daily stress, long working hours, high responsibility, low autonomy and remuneration, among others, particularly when they perform their work in hospital institutions^(3-5)^.

Such circumstances can affect their mental health, cause a drop in their productivity, and increase both absenteeism and physical and mental tiredness, professional exhaustion, anxiety, stress, and depression. Such factors can influence their achievements and their motivation for work, which can decrease the quality of care provided to users under their responsibility^(3,5)^.

Anxiety and stress are among the most often mental disorders in the nursing team. Anxiety is the sixth leading cause of health loss worldwide. In Brazil, about 9.3% of the population is affected, being the country with the highest number of cases in the world^(6)^. Meanwhile, stress is experienced by approximately 90% of the world population and 72% of the Brazilian population that is in the job market suffers some sequel caused by stress; the country ranks second with more cases of stress worldwide, behind only Japan^(6-7)^.

The presence of anxiety and stress among members of the nursing team is expressive and worrying. National and international studies show that these professionals frequently suffer from symptoms of these diseases, proving that they have had some level of anxiety and stress in their work environments, varying between 23 and 73% respectively^(3,5,8-9)^.

For the survey of symptoms related to these conditions, several Likert-type instruments have been used, associating the symptoms reported by the individual^(10-11)^. Another possibility is through salivary compounds or markers, as sources of diagnosis, which have been used in clinics and laboratories, with suitable and accurate results. Therefore, the tracking of these diseases can result in a better prognosis for the person and, with this, the worker can prove mental illness in the medical expertise, as well as avoid/minimize the events of presenteeism, absenteeism, drop in productivity at work and, even to prevent a worsening of the illness^(11-12)^.

Among salivary markers, Salivary Apha Amylase (SAA) is a reliable indicator of sympathetic-adrenal medullary activity. It is one of the main salivary enzymes in humans, produced by the salivary glands under sympathetic stimulation. It can be assessed quickly and non-invasively by collecting saliva and has often been used as a substitute measure for sympathetic arousal. SAA has a positive relationship with adrenergic blocks and β-blockers; its activity is evaluated in stressful situations, which reflect sympathetic activity. Therefore, it can be an effective tool to assess the sympatheticadrenal-medullary system^(13-14)^.

The combination of SAA in the context of clinical assessment of stress and anxiety can contribute to a better understanding of how these disorders participate and influence the individual’s behavioral and cognitive status. This enzyme’s activity, combined with obtaining data through psychological tests, can become a reliable tool for objective assessment of the individual’s mental state, in stressful work environments^(14-15)^.

Given the above and due to the need to seek new knowledge regarding the association between anxiety, stress, and SAA in nursing professionals, the importance and relevance of using this enzyme as a biomarker for assessing anxiety and stress in these workers are justified. Thus, it is believed that SAA can be used in combination with assessment instruments, enabling an early, safe, low-cost, fast, and effective diagnosis, which will allow for more appropriate treatment and anticipating the possibility of better mental health conditions and work to nursing professionals reflecting positively on the quality of nursing care provided to users.

Thus, it was believed that the change in SAA may be associated with anxiety and stress of nursing professionals, as well as anxiety and stress may be associated with other factors. So, the present study aimed to assess whether the change in SAA activity is associated with anxiety and stress in hospital nursing professionals, and whether anxiety and stress are associated with sociodemographic, epidemiological, and occupational factors.

## Method

### Study design

Cross-sectional study, with a quantitative approach, developed in two stages. The first was established for the assessment of anxiety and stress and the second for the collection of saliva and assessment of the participants’ SAA activity. Cross-sectional research is the investigation in which the behavior of one or more variables in groups of subjects at different times is monitored^(16)^.

### Scenario

The study was carried out in a hospital located in a municipality in southeastern Brazil. This institution is a general, philanthropic, and medium-sized hospital. It has 114 beds and attends the Unified Health System (SUS), in addition to private and health insurance services. It has several specialties and serves 95% of its patients through SUS and 5% through other agreements or private individuals. This institution was chosen because it has a population size of compatible nursing workers to answer the objective proposed in this investigation; moreover, it was the city’s largest hospital, with nursing professionals working in the three shifts. Data collection was carried out in 2018, in March and April.

### Participants

The study population consisted of nursing professionals who worked at the hospital in the three work shifts (morning, afternoon, and night), including nursing assistants, technicians, and nurses. Thus, there was a population of 275 professionals, and all were invited to participate, voluntarily, in the study.

To select the participants, the following inclusion criterion was adopted: working at the institution for more than three months. Those who were on sick leave, maternity leave or vacation were excluded; those with a report of some type of cancer, due to the changes that this pathology or its treatment could cause in the activity of SAA^(17)^ and, who used anxiolytics. Thus, the convenience sample consisted of 210 nursing professionals, for the first stage of the study.

After determining SAA activity, samples from participants who were undergoing dental treatment, those who had consumed alcohol or tobacco in the last 24 hours before collection, those who used hormonal drugs, those who had practiced physical activity three hours before collection were excluded. This is because these factors cause changes in SAA activity^(17)^. Thus, 105 nursing professionals participated in the second stage of the study, that is, in the SAA analysis.

### Variables

The study variables were classified into qualitative, continuous, and discrete. The variables used were sex, marital status, alcohol consumption, smoking, physical activity, chronic illness, continuous/daily medication use, professional category, other jobs, anxiety, and stress (qualitative); age, weekly workload, daily workload, and SAA (continuous) and, number of children, monthly family income, time working in nursing, time working at the institution and period/shift/work regime (discrete).

### Data sources/Measurement

In the first stage of the study, three instruments were used. The first was a semi-structured questionnaire with 23 questions on sociodemographic, epidemiological, and labor profile, developed by the researchers, based on national and international literature, which was subsequently subjected to a refinement process with a group of judges, to verify if its items characterized the whole content and allowed to reach the objectives outlined in this research. Next, a pilot test was carried out in another hospital to verify the effectiveness of the instrument, the best approach for data collection and recording, the professionals’ understanding of the questions, as well as to analyze the vocabulary adjustments.

The second instrument was the Lipp’s Stress Symptoms Inventory (LSSI) for Adults, for adults, developed and validated in Brazil by the psychologist Marilda Novaes Lipp, in 1994. This inventory consists of a list of 34 physical symptoms and 19 psychological symptoms, that allow identifying if the person presents stress, what stage of the process he/she is in (alarm, resistance, near-exhaustion, and exhaustion) and if the symptoms are physical or psychological. Thus, the participant must mark on the charts the physical and psychological symptoms he/she has experienced in the last 24 hours, in the last week and the last month^(18)^.

The third instrument used was the Beck Anxiety Inventory (BAI), developed in 1988 by the American psychiatrist Aaron Temkin Beck, translated and validated in Brazil in 2001. This inventory consists of 21 items, which are explanatory statements of the anxiety symptoms. Each symptom is in a line and in the same line there is a row of boxes to be checked, with the statements “Absolutely not; Slightly - it didn’t bother me too much; Moderately - it was very unpleasant, but I could handle it; Severely - I could hardly bear it”. Thus, it is asked how much the person was bothered by each of the symptoms in the past week, within a 4-point Likert scale, with a total score of 0 to 63 points, following the cutoff points: 0 to 10 (within the minimal limit); 11 to 19 (mild anxiety); 20 to 30 (moderate anxiety) and 31 to 63 (severe anxiety)^(9)^.

In the second stage, in which collecting the participants’ saliva took place, the Falcon conical tubes were used, as it has great resistance, temperature stability and for protecting the samples during centrifugation, vortexing and long-term storage in the freezer.

### Data collection

In the first stage of data collection, the professionals’ approach was in the unit itself, and focused on not interfering in the progress of labor activities. The research proposal was presented to them and, after their voluntary approval, they were given an envelope with the Informed Consent Form and the instruments, with a broad explanation of how each was filled out. The instruments were filled out by the participants themselves and returned to the researchers during the work shift.

In the second stage, saliva collection was carried out in three moments (beginning, middle and end of the work shift, at the following times: morning shift – 7 am, 10 am and 12 pm; afternoon shift – 1 pm, 3 pm and 6 pm, night shift – 7 pm, 1 am and 6 am), from all professionals who participated in the first stage of the research, through the Falcon conical tube. That is, each professional had their saliva collected three times. This procedure was performed to verify the variation of the alpha-amylase marker during work activities, as well as its association with anxiety and stress.

To collect saliva samples, participants were informed to avoid using tobacco and alcoholic beverages 24 hours before collection; caffeinated drinks and physical activity in the three hours before collection and food intake and tooth brushing, one hour before collections.

The conical tubes were identified with the codes of each professional, as well as those of the envelopes with the research instruments. They were instructed to spit directly into the tube for approximately 50 seconds or until reaching the 2 milliliters (ml) marker, in an environment of their choice, where they would feel comfortable. These tubes, after each collection, were stored in a Biofreezer at−70ºC. After, all samples were centrifuged at 3,000 revolutions *per* minute (rpm), at a temperature of 4°C for 15 minutes, to separate the supernatant fraction from the saliva^(17)^.

### Analysis plan

The data collected in the study’s first stage were typed into an MS-Excel electronic sheet, version 2010. Then, they were transported to the software Statistical Package for the Social Science (SPSS) version 24, for descriptive and inferential statistical analysis. To assess the reliability of BAI and ISSL, the Cronbach’s alpha coefficient was used. Descriptive analyzes of the variables were performed, using absolute and relative frequency for the qualitative variables and measures of central tendency (mean, median, minimum, and maximum) and dispersion (standard deviation) for the interval variables.

After these analyzes, the Odds Ratio of the independent variables was estimated with the anxiety and stress variables, with the respective 95% confidence interval. To perform the regression analysis, we chose to use logistic regression, given the nature of the dummy variables. The dependent variables of the study were anxiety and stress, both presented dichotomously. The selection of independent variables, which were also dichotomized, was performed using the Bayer method^(19)^.

Thus, all independent variables were included in the analysis. The possible combinations of variables were selected until those with an adjustment to the model were reached. For the final model obtained, the corresponding odds ratios of the parameters were calculated. Next, the sensitivity of the models was assessed using the Receiver Operating Characteristic Curve (ROC curve), observing the Area Under the ROC Curve (AUC) indicators. The sensitivity of the model is assessed according to the accuracy of the diagnosis of a classifier, and its value may vary between zero and one. Thus, an area value below 0.5 or 50% is not valid, while a value equal to 1.0 or 100% is not achieved^(20)^. Also, the fitting quality of the models was assessed using the pseudo-determination coefficient (pseudo R²).

To verify the association between the variables: minimal anxiety *versus* mild, moderate, and severe, with the presence of stress yes *versus* the presence of no stress, Pearson’s Chi-square test was used. The level of significance was set at 5% for all analyzes, that is, the data were statistically significant at *p*<0.05.

In the second stage of the study, to verify the SAA activity, the method of Determination of Salivary Amylase Activity was used in the laboratory. For this, the samples were kept at room temperature (15 to 22ºC), for about 30 minutes. Subsequently, the following protocol was followed: as reagents, ethane sulfonic buffer monohydrate (MES) and 2-chloro-4-nitrophenol-β-D galactopyranosilmaltoside (GAL) for every 5 ml. The saliva samples were diluted 200 times with the MES buffer. This dilution took place in two stages. In the first, 10 µl (Microliter) of saliva was pipetted plus 90 µl of MES in a tube and the contents were mixed in the vortex. In the second, 10 µl of the tube from the first stage was pipetted and an additional 190 µl of MES was added to a second tube. After that, 8 µl of the diluted sample from tube 2 and another 320 µl of GAL were pipetted directly into a microplate well, in duplicate. Thus, an immediate reading was performed on the spectrophotometer at a temperature of 37ºC and 405 nm (Nanometer), using columns^(17)^.

For this analysis, the participants were separated into four groups, according to the results of psychological tests: with stress; with anxiety; without stress and anxiety and, with stress and anxiety, according to the collection times: morning shift – 7 am, 10 am and 12 pm; afternoon shift – 1 pm, 3 pm and 6 pm; night shift – 7 pm, 1 am, and 6 am.

The group “without stress and anxiety” was used as a comparison group. Thus, comparisons were made as follows: no stress and no anxiety *versus* stress; no stress and no anxiety *versus* anxiety and, no stress and no anxiety *versus* stress and anxiety.

To assess the associations between the groups, the data were transported to the GraphPad Prism 5.0^®^ software. First, they were submitted to the ShapiroWilk normality test and, when they accepted a nonparametric distribution, the Mann-Whitney test was applied, considering the median values of SAA. The results were submitted to the Rout Outliers tests. Type I error was set at 5% as statistically significant for all tests (*p*≤0.05).

### Ethical aspects

Based on Resolution 466 of 2012, which deals with research involving human beings, the research project was approved by the Research Ethics Committee, according to Opinion No. 2.528.543 and CAAE 61728016.1.0000.5393.

## Results

About the data found in the first stage of this study of the total of 210 participants, 80.5% were female, 47.6% aged between 30 and 39 years old, 48.6% had partners, 33.3% had no children and had an average monthly family income of R$ 3,041.08 (three thousand and forty-one reais and eight cents) or US$ 789.89 (seven hundred and eighty-nine US dollars and eighty-nine cents). Regarding life habits, 47.1% did not practice physical activity, 22.9% had some chronic disease, 37.1% used medication, 54.3% consumed alcoholic beverages and 88.1% did not smoke. Of chronic diseases, arterial hypertension was the most cited (35.4%); the use of anti-hypertensive, anti-hyper/ hypothyroidism and contraceptive drugs were reported by 25.8% of workers.

It was found that the majority belonged to the category of the nursing technician (80.5%), with experience and time working at the institution of up to 10 years (60.5% and 81.0%, respectively); half of them worked 42 hours a week (50.0%) and 55.2% worked 12 hours or more a day. Regarding the work shift, 40.0% worked on the night shift. Part of them claimed to have another occupation (30.0%); of these, 46.1% worked in another job with a workload of up to 40 hours a week and 41.3% worked up to 6 hours a day.

Table 1 shows the distribution of these workers according to the presence of stress and anxiety levels.

**Table 1:** Distribution of nursing professionals (n=210) according to the presence of stress and anxiety levels. Southeast Minas Gerais, Brazil, 2018

Variables	f	%
**Stress** Yes	122	58.1
No	88	41.9
Total	210	100.0
**Anxiety** Minimal	109	51.9
Mild	57	27.1
Moderate	27	12.9
Severe	17	8.1
Total	210	100.0

When analyzing the presence of stress among nursing professionals, it was found that 58.1% had stress symptoms. Of these, 75.4% were in the resistance phase and 52.5% had psychological symptoms, such as sudden desire to start new projects, decreased libido, excessive tiredness, daily anguish/anxiety, desire to run away from everything, no sense of humor. Regarding the level of anxiety, it was found that the majority had a minimal level (51.9%).

In assessing the internal consistency of ISSL and BAI, using Cronbach’s Alpha, it was considered that there was internal consistency of the instruments, with homogeneity and reliability in their items, since their value was 0.92 and 0.91, respectively.

Table 2 presents the parameters of the Logistic Regression model of the independent variables with anxiety and stress.

**Table 2:** Assessment of the parameters of the logistic regression model of the independent variables with anxiety and stress among nursing professionals (n=210). Southeast Minas Gerais, Brazil, 2018

Variables	Estimate	Standard error	**OR^**^[1]^**^**	**CI 95%^>†^**	p-value
**Anxiety**^‡^ Age range - 20 to 39 years	0.8736	0.3217	2.39	1.27 – 4.50	0.0072
Children - without	-0.6633	0.3232	0.51	0.27 – 0.97	0.0414
Use of medications – yes	0.6398	0.3099	1.89	1.03 – 3.48	0.0402
Weekly workload - over 42 hours	0.6828	0.3001	1.97	1.09 – 3.56	0.0240
**Stress**^§^ Age range - 20 to 39 years	1.3267	0.3285	3.76	1.97 – 7.17	0.0001
Smoker - yes	1.0385	0.5027	2.82	1.05 – 7.56	0.0401
Use of medications – yes	1.0912	0.3337	2.97	1.54 – 5.72	0.0013

^*^OR = Odds ratio; ^†^CI = Confidence Interval (lower/upper); ^‡^Pseudo R² = 8.06%; ^§^Pseudo R² = 9.48%

Concerning the sensitivity of the logistic regression models, the AUC indicators of the ROC curve for the anxiety and stress models were 69.12% and 69.40%, respectively, proving that the models had a good predictive ability.

After analyzing the parameters of all independent variables with anxiety and stress, using the logistic regression model, it was found that the variables age group, children, medication use and weekly workload showed an association with anxiety and the variables age group, smoker, and medication use, were associated with stress, resulting in an adjusted final model (Table 2).

The final model observed that professionals in the age group 20 to 39 years were 2.4 times more likely to have anxiety at higher levels than those aged 40 or over. Workers who did not have children had a protective factor, that is, less chance of having mild, moderate, or severe anxiety compared to those who had children. Besides, professionals on the use of continuous medication were almost twice as likely to experience anxiety at higher levels than those who did not use it. Still, the worker with a weekly workload of more than 42 hours was nearly twice as likely to have anxiety at a mild, moderate, or severe level compared to those who had a workload of up to 42 hours (Table 2).

As for stress, it was also evidenced that professionals in the age group 20 and 39 years were up to 3.7 times more likely to present it compared to those who were 40 years old or more. Those who were smokers were almost three times more likely to have stress when compared to those who were not and workers on the use of continuous medication were almost three times more likely to have it than workers who did not use it (Table 2).

**Table 3:** Bivariate analysis of the association between the anxiety and stress variables among nursing professionals (n=210). Southeast Minas Gerais, Brazil, 2018

Variables	Stress		*p*-value*	OR^†^	CI^‡^ 95%
	Yes	No			
**Minimal Anxiety**	34 (31.2%)	75 (68.8%)	<0.001	1.00	7.34 – 30.35
**Mild, moderate, or severe anxiety**	88 (87.1%)	13 (12.9%)		14.93	

^*^Application of the Person’s Chi-Square Test; ^†^OR = Odds ratio; ^‡^CI = Confidence Interval (lower/upper)

Table 3 presents the bivariate analysis of the association of anxiety with the stress of nursing professionals.

Findings pointed that mild, moderate, and severe anxiety was associated with the stress of nursing professionals (*p*<0.001), that is, the worker who had anxiety at these levels was almost 15 times more likely to experience stress (Table 3).

**Table 4:** Descriptive statistics of Salivary Alpha-Amylase activity by nursing professionals (n=105) according to shifts. Southeast Minas Gerais, Brazil, 2018

Descriptive Statistic	SAA activity (U/ml^*^)								
	Morning shift			Afternoon shift			Night shift		
	**1^st^**	**2^nd^**	**3^rd^**	**1^st^**	**2^nd^**	**3 ^rd^**	**1^st^**	**2^nd^**	**3^rd^**
Mean	48.5	68.59	69.95	35.81	5.82	46.6	30.2	28.59	13.57
Standard deviation	53.45	70.58	80.94	36.3	68.43	45.53	54.2	5.06	34.71
Median	26.97	36.1	47.48	25.76	33.43	28.67	30.2	28.59	13.57
Minimum	2.26	4.68	6.13	7.75	2.42	4.03	3.23	3.55	1.29
Maximum	224.2	304.9	482.7	149.2	261.6	171.5	211.4	216.1	134.9

^*^U/ml = Unit of enzymatic activity

**Figure 1:**
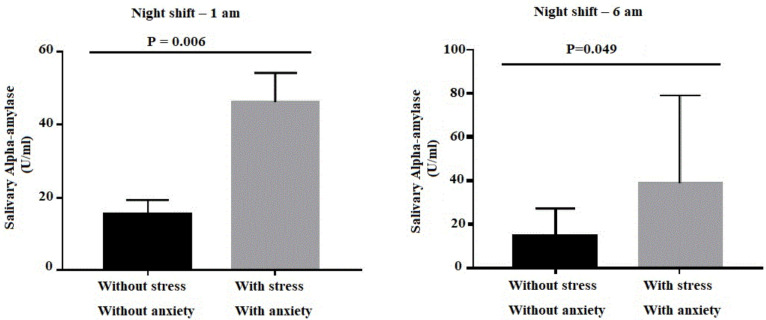
Comparison of the *salivary alpha-amylase* of the “without stress and anxiety” and “with stress and anxiety” groups of nursing professionals (n=105). Southeast Minas Gerais, Brazil, 2018

Regarding data from the second stage of this study, as for the SAA obtained from 105 nursing professionals, when analyzing the enzyme activity in the three collections we could identify that the highest average activity was in the second collection (59.32 U/ml - Unit of enzymatic activity). The third collection showed the highest standard deviation (67.67 U/ml) concerning the others. The highest median was found in the third collection (32.06 U/ml), at the end of the work shift. Among the three collections, the minimum and maximum values of SAA activity were observed in the third collection (1.29 U/ml and 482.7 U/ ml, respectively). Thus, it was possible to observe, on average, that there was an increase in SAA activity in the second collection, that is, in the middle of the work shift and a slight decrease at the end of the shift. Table 4 presents the descriptive data on the SAA activity of nursing professionals according to the work shifts.

When comparing the mean SAA *per* shift, it was found that in the morning the SAA activity was higher in the second collection, with a small increase in the third. In the afternoon shift, SAA activity increased in the second collection and decreased in the third. At night, SAA activity started high and decreased during the work shift (Table 4).

Figure 1 shows the comparison of the median SAA between the group “without stress and anxiety” with the group “with stress and anxiety”, according to the collection times.

When comparing the median SAA value of the groups “without stress and anxiety” and “with stress and anxiety”, between the collection times, we could observe that in the night shift there was a significant association in the hours of 1 am (p=0.006) and 6 am (p=0.049) in the “with stress and anxiety” group. Thus, at these times the SAA activity was greater in professionals who had stress and anxiety. At other times, the median SAA was not significant between groups (*p*>0.05) (Figure 1).

It was also verified, when comparing the groups “without stress and anxiety” *versus* “with stress” and “without stress and anxiety” *versus* “with anxiety”, at the analyzed times, that there was no significant difference concerning the SAA activity (*p*>0.05).

## Discussion

The findings obtained in this study showed that most nursing professionals in the studied hospital had stress and a minimal level of anxiety. Some variables were associated with anxiety (age, children, use of continuous medication, weekly workload) and stress (age, smoking and use of medication). It was found that the SAA activity was higher in nursing professionals who presented with symptoms of stress and anxiety and who worked in the night shift.

This study had some limitations, such as the crosssectional design with convenience sampling, which did not enable to reach all professionals who were not present at the institution but allowed to analyze the saliva of those who were working at that time. Another limiting factor was related to the losses that occurred in the saliva samples, due to the rules regarding the biological analysis protocols. However, even so, it was possible to assess SAA activity in professionals. Still, it was considered as a limitation the fact that the working units of these workers were not controlled/observed, and this may have been a confounding factor for the study.

The results in this research corroborate those of others. In Iran, an investigation carried out with nursing professionals revealed that 68% had medium to high levels of stress and that 31.2% had medium to high levels of anxiety^(5)^. A study carried out in a hospital in the city of Hanoi in Vietnam, with 600 nursing professionals, found that 39.8% of them showed medium to severe levels of anxiety and 18.5% had medium to severe levels of stress^(8)^.

In Brazil, in São Paulo, a survey of 193 hospital nursing workers found that 49.7% of them had more severe levels of anxiety^(21)^. In Espírito Santo, a study also carried out with the hospital nursing team showed that most of them (56.7%) had stress and that half (50.0%) were in the resistance phase, with a higher frequency of physical symptoms^(22)^.

Anxiety is one of the mental disorders that are related to stressful situations from nursing work environments, which, in turn, are linked to work conditions and processes and administrative issues. Other factors such as the lack of resources, work devaluation, the long working hours, the lack of professionals, the performance of tasks in a short time, conflicts between functions and the diversity of units are the main situations that can cause stress and anxiety in nursing professionals^(3,22)^.

The well-being and mental health of nursing workers can be affected by stress and, thus, cause losses in the quality of care, productivity, and efficiency of health care. These professionals work in highly demanding environments, with pressure and several emergencies, which demand fast services. As a result, there is a predisposition to health problems and psychological stress^(5,23)^.

It was also found that some variables were associated with the anxiety and stress of these professionals, including the age group. Therefore, it was observed that the age group between 20 and 39 years were more likely to have anxiety and stress at higher levels, when compared to other age groups.

This variable was also significant in a study with Chinese nursing professionals, showing that younger people had more anxiety and stress. As a result, it was concluded that with increasing age, the symptoms of these disorders decreased^(24)^. In Brazil, a study carried out with professionals from Rio de Janeiro showed a higher frequency of stress at higher levels in those aged between 20 and 40 years old^(25)^.

The experience and maturity of the nursing professional are elements that can help with skills and safety in their activities, as well as in the choice of appropriate coping strategies to stressful situations. As a result, the older the worker’s age and experience, the lower his or her stress level due to work activities^(25)^.

The use of continuous medication was another variable that was also associated with anxiety and stress among nursing professionals. In this study, it was found that those who used medication continuously showed more chance of developing stress and anxiety at high levels.

Studies have shown excessive drug intake among nursing and medical professionals and academics with symptoms of mental disorder. These disorders can be predictive factors for the use of medication to inhibit symptoms, often through self-medication^(26-27)^. The diagnosis and appropriate treatment of mental illnesses, such as anxiety, stress, and depression, can be time-consuming and, at times, uncertain, leading to the worsening of the person’s clinical condition and underreporting of cases. For this reason, some professionals prefer to self-medicate, to minimize the uncomfortable symptoms resulting from the disorder or stressful situation^(27-28)^.

It was also observed in this investigation that workers who did not have children had a protective factor, with less chance of developing anxiety at high levels. Another study pointed out different results from this research, proving that nursing students who had children were less likely to develop some mental disorder, being a protective factor^(26)^. However, the situation of having a child can cause changes in the individual’s life, be it professional or student, as it demands specific care, changes in family routine, concerns about the well-being of the child and the family, besides greater financial expenses^(29-30)^. These factors can favor the onset of anxiety symptoms, especially among women who are in the labor market and often face two or three workdays, as is the case of most participants in this research.

The variable weekly workload was also associated with anxiety. The worker who worked more than 40 hours a week was more likely to have higher levels of anxiety. A review study carried out with nursing professionals showed that long working hours are among the main factors that cause anxiety and depression^(31)^. Research carried out with nursing professionals at a public hospital in southeastern Brazil found a positive relationship between weekly workload and the onset of some mental disorders, such as anxiety and stress^(22)^. Long working hours can be considered stressful. Someone who works many hours, with double or triple workdays, has less time for rest and leisure; these factors can cause fatigue, physical and mental exhaustion, besides disorders such as anxiety and stress^(32)^.

The use of tobacco by the participants was also another variable that was associated with stress, pointing out that those who were smokers were more likely to have stress. Other studies have also demonstrated this result, highlighting that tobacco has been used to deal with stressful situations, as the cigarette has been used as a relief from daily tensions^(33-34)^.

In this work, it was found that mild, moderate, and severe anxiety was associated with the professionals’ stress. That is, the worker who had anxiety also had a greater chance of having stress. Studies carried out with nursing professionals in Hong Kong and Iran, also showed a positive correlation between anxiety and stress symptoms among them^(5,24)^.

These correlations were also pointed out by another study, which evidenced that the exhaustive and unhealthy work environment appears as the main factor causing stress, simultaneously with other psychic symptoms, such as anxiety^(35)^. Anxiety and stress can impair the efficiency, productivity, and quality of healthcare. Thus, it is suggested the need to create preventive strategies against stress and anxiety, as well as for the rehabilitation of professionals with psychological distress in the work environment^(5)^.

When observing the SAA activity of nursing professionals in the “with stress and anxiety” group, it was observed that there was a significant association with the night shift, at 1 am and 6 am. It was found that at these times, that is, in the middle and at the end of the work shift, the SAA activity was greater in professionals who had stress and anxiety.

The results of this study corroborate with other investigations, which also pointed to an association between SAA and psychological factors. In a survey conducted with university athletes in Korea, a positive correlation was identified between anxiety and the increase in SAA activity, demonstrating that there may be correlations between psychological anxiety and physiological anxiety factors, which can cause a decrease in athlete’s performance^(36)^. In an investigation carried out with workers from different occupational categories in Canada, the relationship between SAA activity and various psychosocial characteristics related to work was highlighted. The study indicated that psychological distress was associated with increased SAA, with the afternoon shift, between 2 pm and 4 pm, the period of greatest SAA activity^(37)^.

Another survey of children undergoing dental treatment in Saudi Arabia found that patients’ phobia and anxiety were associated with higher levels of SAA^(38)^. In an investigation conducted with medical students in China, it was found that procrastination and perceived stress were associated with higher levels of SAA^(39)^.

The variation in SAA can be a biological indicator of exposure to stressors within the workplace. Work characteristics such as psychological demands, lack of support from the team, interpersonal conflicts, work appreciation, psychological suffering, job insecurity, are stressors capable of triggering a physiological response in the body, which can be assessed changes in SAA^(37)^.

As demonstrated, studies have shown significant associations between SAA and psychological factors, such as stress and anxiety^(35-39)^. Therefore, it is believed that SAA can be a promising biological marker of stress and anxiety in nursing professionals, as shown in the present work. However, there is a need for further investigation using this enzyme, to establish reference values, as well as standardization for its use as a diagnostic tool in assessments to psychological factors and the world of work. Thus, it is emphasized that there is a need for greater awareness of the health of these workers, since stress and anxiety can cause damage^(5)^, risking both the health of those who work and the quality of care provided to the user at health services.

This study provides important information to science and health promotion for nursing professionals. This, by indicating SAA as a possible biomarker of stress and anxiety in these professionals. In this way, with the assessment of SAA activity, a more accurate and quick diagnosis can be provided to workers, which will enable appropriate treatment and the possibility of better mental health and work conditions, reflecting positively on the quality of nursing care.

## Conclusion

Most nursing professionals participating in this study had stress and anxiety. Some variables were associated with professional anxiety and stress. SAA activity was shown to be significantly higher in professionals who experienced stress and anxiety, at certain periods of observation and work shift. These results, in line with those found in other studies mentioned in this investigation, indicate that this enzyme can be a promising biomarker of stress and anxiety.

The most advanced technologies for the diagnosis and monitoring of mental illnesses offer more readiness and confidence to validate and watch the improvement of individuals during treatment. Furthermore, they contribute to commonly assess workers with highly stressful activities, as is the case with nursing professionals, aiming at preventing injuries associated with stress and anxiety.
